# Determinants of length for age Z scores among children aged 6–23 months in Central Java, Indonesia: a path analysis

**DOI:** 10.3389/fnut.2023.1031835

**Published:** 2023-04-17

**Authors:** Martha Irene Kartasurya, Ahmad Syauqy, Suyatno Suyatno, Julian Dewantiningrum, Nuryanto Nuryanto, Sunarto Sunarto, Muflihah Isnawati, Yusi Dwi Nurcahyani, Erna Kusuma Wati, Pramesthi Widya Hapsari, Mohamad Samsudin, Noviati Fuada

**Affiliations:** ^1^Faculty of Public Health, Universitas Diponegoro, Semarang, Indonesia; ^2^Faculty of Medicine, Universitas Diponegoro, Semarang, Indonesia; ^3^Nutrition Department, Poltekkes Kemenkes Semarang, Semarang, Indonesia; ^4^Research Services, Center for Health Research and Development, Magelang, Indonesia; ^5^Faculty of Health, Universitas Jenderal Soedirman, Purwokerto, Indonesia; ^6^Research Center for Public Health and Nutrition, National Research and Innovation Agency (BRIN), Cibinong, Indonesia; ^7^Organization for Health, National Research and Innovation Agency, Jakarta, Indonesia

**Keywords:** length for age Z-scores, stunting, under two children, path analysis, Central Java, Indonesia

## Abstract

**Introduction:**

Length for Age Z (LAZ) score determinants are complex and vary among different areas, but it is important for designing effective and efficient strategies to decrease stunting prevalence among children under 2 years. This study aimed to investigate the determinants of LAZ scores among children under 2 years in Central Java, Indonesia.

**Methods:**

This study was conducted on the 2021 Indonesian Nutritional Status Study (INSS) dataset, which was a cross-sectional survey. Data on 3,430 children aged 6–23 months from Central Java province were derived from the 2021 INSS data. After missing data elimination, 3,238 subjects were included in the analysis. Determinant factors included direct and indirect factors. Direct factors were the mother's age, birth weight Z score (BWZ), birth length Z score (BLZ), exclusively breastfed history, dietary diversity scores (DDS), empty calorie drink consumption, unhealthy snacks consumption, and infections. Indirect factors were early initiation of breastfeeding (EIBF) and *posyandu* (integrated health post) utilization. Underlying factors were socioeconomic status (SES) and the mother's education. Bivariate analyses and multiple linear regressions were conducted. A path analysis with a hypothesized model based on the UNICEF conceptual framework was also performed.

**Results:**

Stunting, wasting and underweight proportions among the subjects were 19.1%, 7.6% and 12.3%, respectively. The mean LAZ scores were −0.95 ± 1.22; the mother's age was 29.7 ± 5.95 years; BWZ was −0.47 ± 0.97; BLZ was −0.55 ± 1.05; and DDS was 4.45 ± 1.51. The infection proportion among the subjects was 28%. BWZ and BLZ were positively correlated to LAZ scores, with r = 0.267 (*p* < 0.01) and r = 0.260 (*p* < 0.01), respectively. The mother's age was negatively correlated to LAZ scores with r = −0.041 (*p* < 0.05). Maternal education was positively correlated to SES but had no direct effect on LAZ scores. LAZ score determinants of BLZ (*p* < 0.001) and SES (*p* < 0.001) showed positive direct associations with LAZ scores, but the mother's age (*p* = 0.039), exclusively breastfed history (*p* < 0.001), and empty calorie drinks consumption (*p* < 0.001) had negative associations with LAZ scores.

**Conclusion:**

To prevent stunting among children aged 6–23 months in Central Java, Indonesia, intervention programs to increase the nutritional status of women at child-bearing age and nutrition education on child feeding practices should be conducted more efficiently and effectively.

## 1. Introduction

Stunting prevalence among children under 5 years in Indonesia is still high, reaching 24.4%, while the prevalence in Central Java is 20.9% (1). Although the prevalence of stunting in Central Java has decreased compared to the prevalence in 2019, it still has to be reduced further because Central Java's dense population of approximately 35 million in 2020 resulted in a big absolute number of stunted children. Central Java is the third populated province on Java Island, which is the most populous island in Indonesia, with 152.4 million inhabitants. Thus, measures should be taken to decrease the prevalence of stunting in this province.

Stunting development can be started in the first 1,000 days of life, from conception until 2 years old. Thus, the problems that occurred during pregnancy, the exclusive breastfeeding period, and the complementary feeding would affect the linear growth (2). The LAZ scores then slow down at 2 to 3 years old with no catch-up period (3). Stunted children are unlikely to catch up to their peers. Thus, possible interventions may only be effective in the first 2 years of life (4). Since 2006, the World Bank recommends focusing on providing nutrition support during the window of the opportunity period, which begins at conception and ends at 2 years of age (5). This period is a critical time when children undergo rapid physical and brain growth and development, so nutrition adequacy is very important (6). Stunting reduction should be more focused on this group through various specific and sensitive interventions so the young population in the future will not be a burden but will be more productive (7–9).

Risk factors of stunting could be different in each area, based on their characteristics. As stunting is the result of linear growth problems, determinants of LAZ scores among children in Central Java should be assessed to have the correct measures in stunting prevention and handling. Determinants of LAZ scores include direct, indirect, and underlying factors. Direct factors consist of nutrition intake, infection, and maternal factors. Indirect factors cover food accessibility, childcare, and environmental health. Underlying factors are social economic status, cultural, and political conditions (10). A previous study showed how diverse the factors were which influenced stunting in each province, regency, and even subdistrict (11). Therefore, the analysis of determinants should use a multilevel method such as path analysis. With path analysis, the pathway of factors and the partial and total effects can be measured.

In this study, the magnitude of direct, indirect, and underlying factors available from the 2021 INSS data in determining LAZ scores was calculated through a path analysis model. Path analysis evaluates associations between a dependent variable and two or more independent variables. With the known magnitude, preventive measures can be taken more specifically and properly based on local problems. Therefore, this study aimed to investigate the determinants of LAZ scores among children under 2 years in Central Java, Indonesia.

Since 2019, the Indonesian Ministry of Health has started an annual Indonesian Nutritional Status Study (INSS or SSGI), which was conducted in all provinces in Indonesia. This study used the 2021 INSS data, especially data on children aged 6–23 months in Central Java. Data on infants aged 0–6 months old was not used because the analysis include dietary diversity score data, which is only available for infants who were not exclusively breastfed. Therefore, we excluded the infants aged < 6 months to prevent selection bias.

## 2. Materials and methods

This study was conducted on the 2021 Indonesian Nutritional Status Study (INSS 2021)/SSGBI 2021 data collected annually since 2019 (1). Data were collected in January–December 2021 using two different questionnaires, i.e., household and individual levels. Data collection was done in a cross-sectional survey, using stratified two-stage sampling, with the population of households that had children under 5 years. The samples were children under 5 years in 1,53,228 households from 14,889 census blocks of the 2021 Indonesian National Socio-economic Survey (SUSENAS) in 34 provinces and 514 regencies/cities.

**Figure 1:**
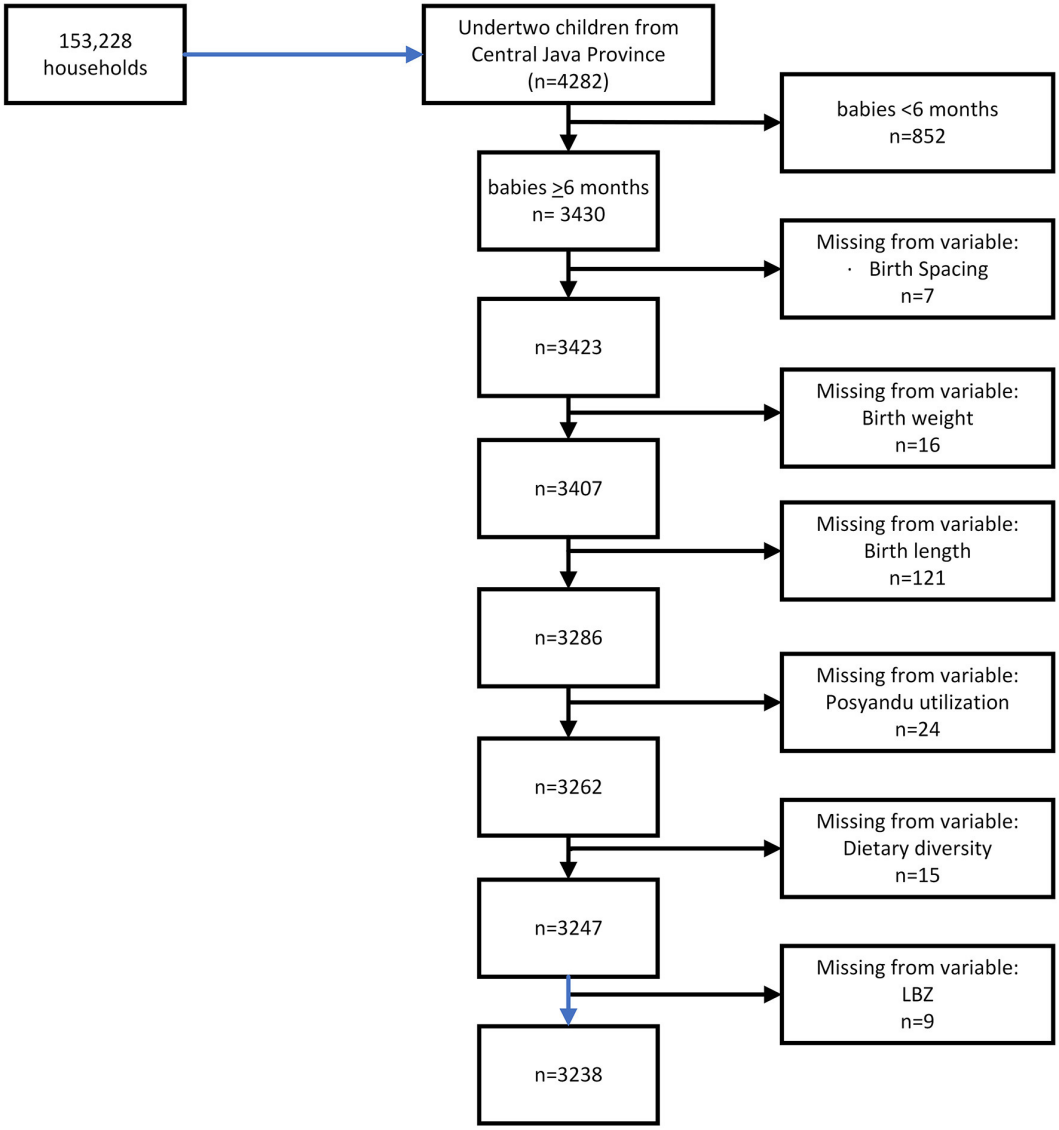
Sample determination.

In this study, only data on children aged <2 years from Central Java province were used (*n* = 4,282). Then we chose children aged 6–23 months (*n* = 3,430), and after missing data deletion (*n* = 192), the complete analysis was performed on 3,238 children (Figure 1).

The dependent variable was the LAZ scores which were counted based on the WHO anthropometric calculator on a continuous scale. All extreme values of LAZ > 6.0 and LAZ < −6.0 were excluded. The independent variables were direct, indirect, and underlying determinants of LAZ scores available from the 2021 INSS data. The direct determinants of LAZ scores include those as follows: (1) maternal factor: the mother's age; (2) child factors: birth weight Z scores (BWZ) and birth length Z scores (BLZ); (3) food consumption: dietary diversity score, exclusively breastfed history, unhealthy snack food consumption, and empty calories drink (starch/ sugary water) consumption; and (4) infection. The indirect determinants of LAZ scores include early initiation of breastfeeding (EIBF), *posyandu* utilization, drinking water source, and sanitation. The underlying determinants of LAZ scores include the mother's education and socioeconomic status.

Data on all variables were gathered from the interviews and observations to the records available in the Maternal and Child Health Handbook, or the other available records. If the record was not available, interviews with the mothers were done based on the subjects' memory. The mother's age was defined as the number of years the mother was alive until the interview time. Birth weight and birth length were measured by the midwives at deliveries and usually were recorded in the Maternal and Child Handbook/other record forms such as the mother's cohort book. At the survey, the birth weight and birth length were observed from the record or mothers'/other family members' memories. Thereafter, birth weight (BWZ) and birth length Z scores (BLZ) were calculated by the researchers based on WHO ANTHRO software.

A dietary diversity score was defined as the number of food groups consumed in the last 24 h by the subjects. The food groups were those as follows: 1. breast milk; 2. grains, roots, tubers, and plantains; 3. pulses (beans, peas, lentils), nuts, and seeds; 4. dairy products (milk, infant formula, yogurt, and cheese); 5. flesh foods (meat, fish, poultry, and organ meats); 6. eggs; 7. vitamin-A-rich fruits and vegetables; and 8. other fruits and vegetables. The minimum dietary diversity score is 5 (12). Unhealthy snack consumption was defined as the consumption of extrudate snacks (flavored ball puffs of starch, crisps, etc.) in the last 24 h. Empty calorie drink consumption was defined as the consumption of starch water, honey, tea, coffee, sugary water, fruit juice, and milk-flavored sweet thick water in the last 24 h.

Early breastfeeding initiation was defined as the baby directly put on the mother's breast at the 1st h of delivery without any barrier (12). Infants, as mothers reported, who first received food or liquids other than breastmilk after 6 months were categorized as having exclusively breastfed history (EBF history). Infants, as mothers reported, who first received other food or liquids before 6 months were categorized as having a non-exclusively breastfed history (non-EBF history).

Infection was defined as the existence of at least one of these infections experienced by the subject: diarrhea, upper respiratory tract infection (URTI) in the previous month, or pneumonia, tuberculosis, measles, and worms' infections in the last 6 months. All of the infections were defined based on the health personnel's diagnosis or symptoms experienced by the children which were noticed by their mothers. In the analysis, infection was used as a nominal variable without considering the kind of infection: the existence or no existence of infection.

*Posyandu* is an integrated health post, which is organized by the local community. *Posyandu* activities include growth monitoring, nutrition education, basic healthcare services such as diarrhea treatment, family planning services, and immunization of children under 5 years. The basic healthcare service is provided by the professional health worker, but for the other services, health cadres from the community serve voluntarily. In general, the level of participation is relatively high, as *posyandu* is the activity by, for, and from the local community (13). *Posyandu* utilization was defined as the mothers' or child caregivers' attendance at the integrated health post. The drinking water source was the source of water for drinking purposes in the household. Proper drinking water was the saved drinking water which includes tap water, public tap water, public hydrant, water terminal, rainwater storage, water springs, protected well, artesian well, or pump well which have a distance of more than 10 m from the garbage storage. Bottled water, water from the mobile vendor, water sold from a water tank, and non-protected water spring/well were not included as proper drinking water (14). Sanitation was the sanitation facilities available, including the availability and type of toilet and the sewage system. The proper sanitation facility was the toilet which fulfills health requirements, completed by goose' neck closet, and the final disposal place was a septic tank or wastewater treatment installation. This facility can be used by the household only or by sharing the facility with other households (15).

The mother's education was defined as the highest level of education which has been passed by the mothers, such as elementary school, junior high school, high school, undergraduate, and postgraduate levels. The group of mothers who had no education at all was combined with the group of mothers who had not completed elementary school as they were only 0.3%. Socioeconomic status was defined as the wealth quintiles based on durable goods possessions, which have been used by the Indonesian National Bureau of Statistics in many national surveys.

For statistical analysis, frequencies and percentages were used to describe the categorical variables, while means and standard deviations were used to describe the continuous variables. LAZ scores, the mother's age, WBZ, LBZ, and dietary diversity score were normally distributed. The other determinant variables were measured as categorical variables. Correlation tests were used to assess the associations between LAZ scores, the mother's age, birth weight Z scores, birth length Z scores, and dietary diversity scores. The results are provided in **Table 2**. As the correlation between DDS and LAZ was influenced by sex and age, a generalized linear model (GLM) which included sex and child's age as the covariates were run. The final GLM model to examine DDS and LAZ correlation was only controlled by the child's age as sex was not a significant covariate. The summary of the result was added as a note in **Table 2**. We examined the independent associations between each predictor and LAZ scores and dropped any variable that was not associated at p < 0.05. The results can be seen in **Table 3**. As WBZ and LBZ scores were correlated at *r* = 0.593, we decided to include only LBZ scores as the determinants of LAZ scores. In the next step, we included the significant determinants in a multivariate model of each factor group (child factors, food intakes, environmental factors, socioeconomic status, and the mother's education) to predict LAZ scores and dropped any variable that was not associated at *p* < 0.05 (16). The results can be seen in **Table 3**. All of these processes were performed using IBM SPSS version 25 (SPSS Inc., Chicago, IL, USA).

**Figure 2:**
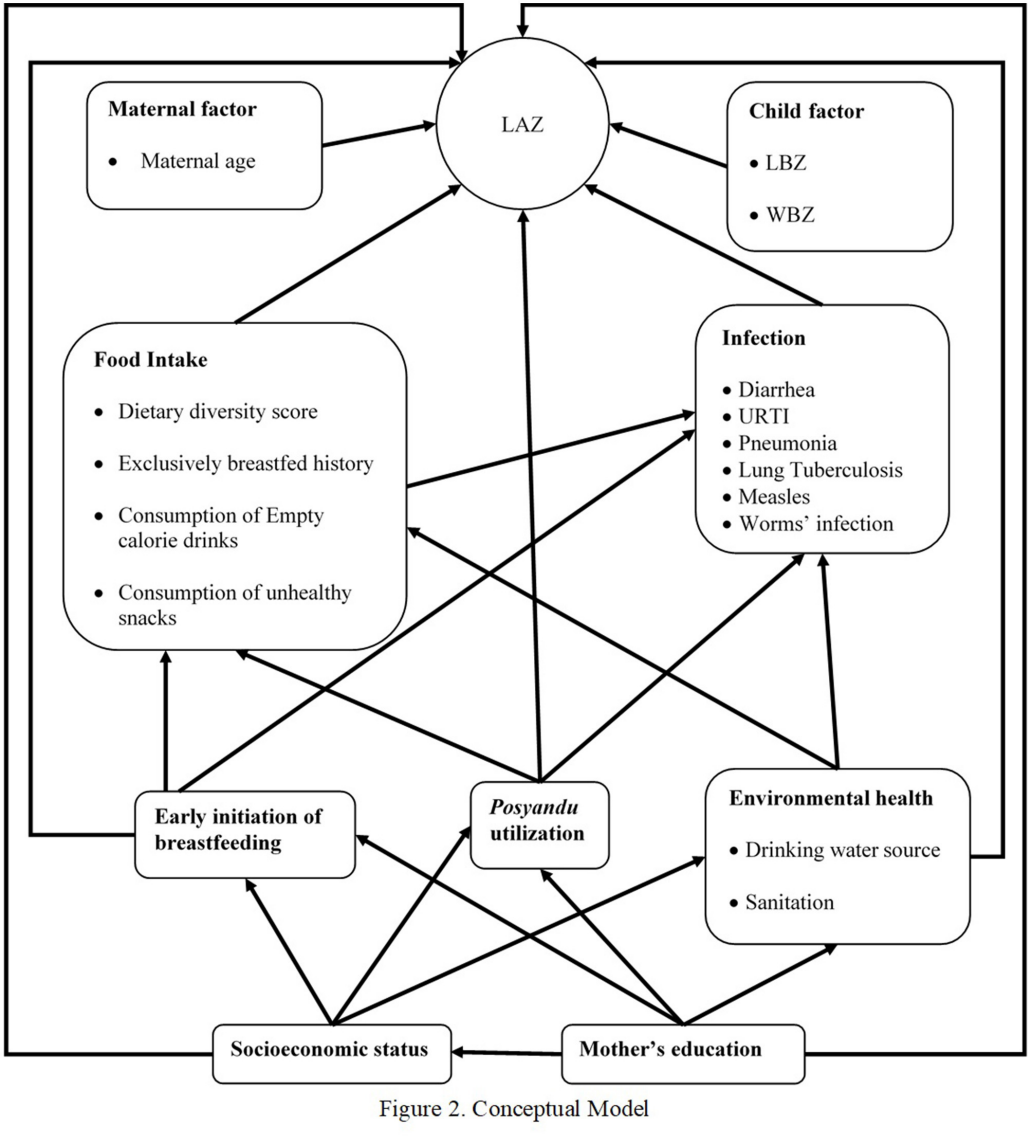
Conceptual model.

**Figure 3:**
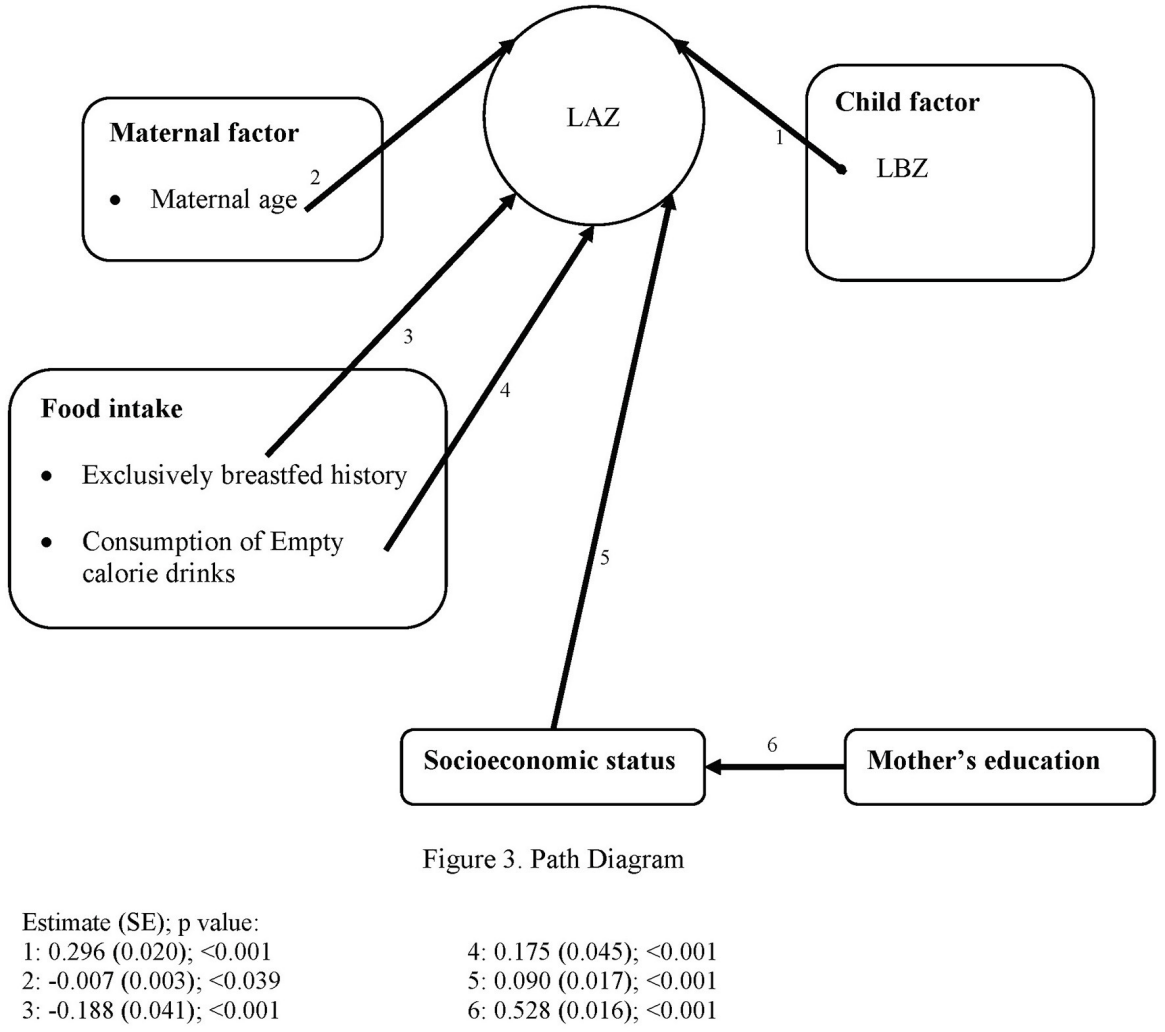
Path diagram.

The analysis was then continued with a path analysis model in SPSS AMOS based on the conceptual model in Figure 2. This figure was based on the UNICEF conceptual framework for undernutrition. In this figure, the direct determinants for LAZ scores were grouped as maternal and child factors, food intake, and infection. The indirect factors included EIBF, *posyandu* utilization, and environmental health, while the underlying factors were socioeconomic status and the mother's education. All of the arrows showed the correlations, which were analyzed, directly to LAZ scores and through the intermediate variables. The analysis using this conceptual model was continued after the previous steps for all of the retained variables. Then, all of the insignificant paths (p ≥ 0.05) were dropped. The final model can be seen in Figure 3, and this model fulfills all the goodness of fit indicators (NFI-CFI > 0.9 and RMSEA < 0.05).

## 3. Results

Table 1 shows the descriptions of the characteristics, direct, indirect, and underlying factors of LAZ scores. It is shown that the mean and standard deviation of subjects' LAZ scores were −0.95 ± 1.22, which means they were in a relatively low stature based on the WHO ANTHRO standard. The proportion of stunted children was 19.1%, wasted 7.6%, and underweight 12.3%. The mean and standard deviation of mothers' age was 29.73 ± 5.95 years; the percentage of mothers aged < 20 years was only 2.1%; and those aged >35 years was 18.3%. The mean WBZ and LBZ were −0.47 and −0.55, respectively. The mean dietary diversity score was 4.45, with the proportion of children who met the minimum dietary diversity was 50.2% (12). All other descriptions of the determinant variables are shown in Table 1. Among the subjects, 6.5% (*n* = 210) were born as low birth weight babies based on the category of < 2,500 g.

**Table 1:** Descriptions of characteristics, direct, indirect, and underlying factors of LAZ scores among children aged 6–23 months old in Central Java province based on INSS 2021 (*n* = 3,238).

	**n**	**%**	**Mean**	**SD**
LAZ score			−0.95	1.22
Stunted	617	19.1		
WHZ score			−0.40	1.20
Wasted	248	7.6		
WAZ score			−0.78	1.14
Underweight	398	12.3		
**Child Factors**				
BLZ scores			−0.55	1.05
Stunted at birth	272	8.4		
BWZ scores			−0.47	0.97
Low birth weight	166	5.1		
**Maternal Factors**				
Mother's age (years)			29.73	5.95
< 20 years	69	2.1		
20–35 years	2,577	79.6		
>35 years	592	18.3		
**Food Intake**				
Dietary diversity (score)			4.45	1.51
Meet the Minimum Dietary Diversity (MDD)	1,624	50.2		
**Exclusively breastfed history**				
Yes	1,749	54.0		
Consumption of Empty calorie drinks (starch water, honey, tea, coffee, sugar water, fruit juice, sweetened condensed milk, etc.)				
Yes	984	30.4		
Consumption of unhealthy snacks: extrudates in the forms of puffs, such as cheese flavored balls, crisps, etc.)				
Yes	1,753	54.1		
Infection (experienced at least 1 out of these infections: diarrhea, URTI in the previous month, or pneumonia, tuberculosis, measles, worms' infection in the last 6 months)				
Yes/ having infection	908	28.0		
**Early initiation of breastfeeding**				
No	1,920	59.3		
***Posyandu*** **utilization**				
Not utilized *posyandu* in the last year	164	5.1		
**Environmental Factors**				
**Drinking water source**				
Not proper	1,436	44.3		
**Sanitation**				
Not proper	421	13.0		
**Socioeconomic status**				
Lowest 20%	294	9.1		
Low Middle	780	24.1		
Middle	798	24.6		
High Middle	801	24.7		
Top 20%	565	17.4		
**Mother's education**				
Did not finish Elementary School	57	1.8		
Completed Elementary School (Grade 1–6)	664	20.5		
Completed Middle School (Grade 7–9)	998	30.8		
Completed High School (Grade 10–12)	1,132	35.0		
Completed Diploma 1–3	121	3.7		
Completed Bachelor or master or doctoral degree	266	8.2		

Table 2 shows that the variables correlated with LAZ scores were the mother's age, BWZ, BLZ, and dietary diversity score. The mother's age (*r* = −0.041, *p* < 0.05) and dietary diversity score (*r* = −0.036, *p* < 0.05) were negatively correlated with the LAZ scores, while BWZ and BLZ were positively correlated with the LAZ scores. Then, the correlation between DDS and LAZ scores was controlled for sex and age in a GLM. As child's age was the only significant confounder, this variable was the only covariate included in the GLM. After adjustment for child's age, the correlation between DDS and LAZ was no longer significant (*p* = 0.079) with a positive parameter estimate of 0.026. In the same model, the child's age negatively correlated to LAZ with a parameter estimate of −0.049 and a *p* < 0.001. The mean LAZ of the children who met the minimum dietary diversity (DDS ≥ 5) (12) was −0.99 ± 1.198, while the mean LAZ of the children who did not meet the minimum dietary diversity (<5) was −0.92 ± 1.258. The independent *t*-test results showed no difference in LAZ between the groups, with a *p*-value of 0.114. BWZ was strongly correlated with BLZ, and dietary diversity score was positively correlated with the mother's age.

**Table 2:** Correlations between continuous variables (*n* = 3,238).

	**LAZ scores**	**Mother's age**	**Birth length Z scores**	**Birth weight Z scores**	**Dietary diversity (score)**
LAZ scores	1				
Mother's age	−0.041^*^	1			
Birth length Z scores	0.260^**^	−0.029	1		
Birth weight Z scores	0.267^**^	0.005	0.593^**^	1	
Dietary diversity (score)	−0.036^*^	0.035^*^	0.013	−0.011	1

* p < 0.05.

** p < 0.01.

After controlled for the child's age, the correlation between DDS and LAZ was no longer significant (p = 0.079).

Table 3 shows the variable selection results based on the independent association to LAZ scores and adjustment to the other variables in the same category. As BWZ and BLZ were strongly correlated to each other (*r* = 0.593), we decided to include BLZ only in the model. Based on the *p* ≥ 0.05 of the independent correlation to LAZ scores, these variables of unhealthy snack consumption, infection, EIBF, *posyandu* utilization, and drinking water source were dropped from the model. After the adjustment of the other variables in the same category, the dietary diversity score dropped as this variable no longer had a significant effect on LAZ scores.

**Table 3:** Variable selection results.

	**Independent association with LAZ**			**Adjusted for other variables in the same category**		
	**Estimate (SE)**	***P*** **value**	**Decision**	**Estimate (SE)**	***P*** **value**	**Decision**
**Child Factors**						
LBZ	0.30 (0.02)	<0.0001	Retain	0.30 (0.02)	<0.0001	Retain
**Maternal Factors**						
Mother's age (years)	−0.008 (0.004)	0.019	Retain	−0.008 (0.004)	0.019	Retain
**Food Intake**						
Dietary diversity (score)	−0.03 (0.1)	0.040	Retain	−0.01 (0.01)	0.353	Drop
Exclusively breastfed history	−0.16 (0.04)	<0.0001	Retain	−0.16 (0.04)	<0.0001	Retain
Consumption of Empty calorie drinks (starch water, honey, tea, coffee, sugar water, fruit juice, sweetened condensed milk, etc.)	0.18 (0.05)	<0.0001	Retain	0.18 (0.05)	<0.0001	Retain
Consumption of unhealthy snacks: extrudates in the forms of puffs, such as cheese flavored balls, crisps, etc.	0.08 (0.04)	0.060	Drop	-	-	-
Infection	−0.07 (0.05)	0.125	Drop	-	-	-
Early initiation of breastfeeding (EIBF)	0.03 (0.04)	0.532	Drop	-	-	-
*Posyandu* utilization	−0.11 (0.10)	0.244	Drop	-	-	-
**Environmental Factors**						
Drinking water source	−0.05 (0.04)	0.238	Drop	-	-	-
Sanitation	0.19 (0.06)	0.003	Retain	0.19 (0.06)	0.003	Retain
Socioeconomic status	0.11 (0.02)	<0.0001	Retain	0.11 (0.02)	<0.0001	Retain
Mother's education	0.08 (0.02)	<0.0001	Retain	0.08 (0.02)	<0.0001	Retain

The conceptual model of the path analysis is shown in Figure 2, and the results of the path analysis model are shown in Figure 3. This figure shows only the significant pathways of determinants. The goodness of fit results of NFI 0.954, RFI 0.913, IFI 0.965, TLI 0.934, CFI 0.965, and RMSEA 0.030 (0.022–0.038) showed that this is a good fit model. There were five direct determinants of LAZ scores: LBZ, the mother's age, exclusively breastfed history, consumption of empty calorie drinks, and socioeconomic status. The mother's education was not a direct determinant of LAZ scores but an indirect determinant through the intermediate factor of socioeconomic status. LBZ and socioeconomic status showed a positive estimate, which means that the subjects with higher LBZ and socioeconomic status had higher LAZ scores. The mother's age and exclusively breastfed history were negatively associated with LAZ scores, which means that the older mother's age had the lower LAZ scores and the subjects who had exclusively breastfed history had the lower Z scores. The subjects who consumed empty-calorie drinks had lower LAZ scores.

## 4. Discussion

The prevalence of stunting among children aged 6–23 years old in this study was lower than among children under 5 years old in this 2021 INSS result (24.4%) (1). Thus, many factors still influence the HAZ scores in children after 2 years, resulting in stunting among children under 5 years. However, since the window opportunity for intervention is in the first 1,000 days of life, it is important to set the determinants of LAZ scores among children under 2 years. Few studies had been conducted at this age (17, 18). The determinants of LAZ scores among children under 2 years could differ from the factors for LAZ scores among children under 5 years (19, 20). The results of this study can be used for the prioritization of stunting prevention and alleviation programs based on the total effect of determinants found in this study.

In this study, it was found that the mother's age was related to LAZ scores among children under 2 years. The older the mother, the lower the LAZ scores. Other studies have shown that the mother's age <20 years had a higher risk of having a stunting child (21–23). In this study, only a small percentage of the subjects (2.1% or 69 subjects) were <20 years old while 18.3% were >35 years old. Thus, the mothers who were older than 35 years had more influence on the results. A study in Nepal showed a consistent result with this study (24). Older mothers tended to have a lower nutrient deposit than younger ones as they already had some children.

The child factor of BLZ is strongly related to LAZ scores. An increase in 1 SD in BLZ raised LAZ scores by 0.296. BLZ was the strongest determinant of LAZ scores among children under 2 years in this current study. Birth weight and birth length were interrelated. Logically, the shorter babies will have a lower birth weight. A study in Northern Ghana showed that the low birth weight babies had lower HAZ scores of −0.98 compared to the normal birth weight among children at 6–59 months old (25). Christian et al.'s study in 2013 indicated that low birth weight was associated with 2.5–3.5-fold higher odds of stunting (26). This study also showed that the low birth weight was caused by small for gestational age (SGA), which had a 20% attributable risk for childhood stunting.

After controlling for the child's age, DDS was no longer correlated to LAZ scores. The child's age was negatively correlated to LAZ scores; the older the children, the lower the LAZ scores. This finding was similar to some other studies which showed that the older children had lower Z scores (27, 28). In this study, the older children also consumed more varieties of food; thus, the child's age confounded the correlation between DDS and LAZ scores.

Consumption of sugary water, starch water, and other empty calorie drinks was also related to a lower LAZ, which means that the children who were fed these kinds of drinks had a lower LAZ score of 0.18. Empty-calorie drink consumption replaced the money that should have been used for nutritious food and replaced the food volume in the children's stomachs. By consuming the empty calorie drinks, the children had reached satiety, but the acquired nutrients were very limited (29). Furthermore, the consumption of sugary drinks and foods has a negative effect and causes dental caries, pain, and eating difficulty, resulting in an inadequate intake of nutrients (30, 31).

Surprisingly, in this study, exclusively breastfed history results in lower LAZ scores. The subjects who had a history of exclusively breastfed had 0.19 LAZ scores lower than those who did not. This condition was not related to socioeconomic status as the lower LAZ scores for the children who had a history of exclusively breastfed appeared in all levels of socioeconomic status. This study was performed in a cross-sectional design, and the subjects who had exclusively breastfed history might have continued to be fed by the low quality of complementary food, which then would result in low LAZ scores. Furthermore, by the definition of exclusively breastfed history (got the first food/drink other than breastmilk at ≥6 months), we might have included the subjects who have had prolonged exclusive breastfeeding, which might have resulted in nutrient inadequacy and led to a lower LAZ. Unfortunately, we did not have any data on another variable on the delayed introduction of complementary foods. Thus, the exclusively breastfed history was not the cause of low LAZ but just the history of exclusive breastfeeding will not be enough to prevent stunting in children under 2 years (32). In this study, the children under 2 years who were previously exclusively breastfed have a higher risk of stunting. This fact differs from another study in East Indonesia that showed exclusive breastfeeding is a protective factor against stunting (33).

Data from Central Java health profile 2021 available on the website showed that the prevalence of exclusive breastfeeding was 72.5 %, which increased from 67.3 % in 2020 (34). However, these data were collected not on the children at 6 months but on any age at the time of the children were surveyed. It is possible that after the survey, the children were then given other food/drinks than breastmilk before 6 months. Our results showed that the number of children with a history of exclusive breastfeeding was 54.0%. Thus, our data on EBF history were reasonable.

Another consideration was the influence of the low nutritional status of the mothers, which then might have modestly influenced the breast milk content (35). A study in Indonesia indicated that lactating women had a low intake of micronutrients, which then modestly affected the micronutrient content of breast milk (36). This modestly lowers the content of micronutrients in breast milk that may have affected the micronutrient adequacy of their infants. A study in Bogor Indonesia showed that the lactating mothers' nutritional status was lower than the pre-pregnant women's nutritional status (37). Another study in Indonesia also showed that the nutritional status of lactating women was lower compared to the women who were not lactating (38). Fikawati et al.'s study in Java Island, Indonesia, also showed that the energy consumption of lactating women was low (39). Unfortunately, data on mothers' nutritional status from the 2021 INSS that can explain the results were not available. Thus, this explanation was unlikely to fully explain the finding on the negative correlation between the history of exclusive breastfeeding and LAZ scores.

The other possible explanation of this negative correlation was the reverse causality, in which the children who were having poor growth as a result of poor diet and infections tended to be breastfed or prolonged exclusively breastfed (40). Unfortunately, there was no specific data on children's diet or breastfeeding status after 6 months. Furthermore, data on infection was only cross-sectional. Non-standard definitions of infant feeding practices that were used in this study could also be a potential problem to the finding of this negative correlation between the history of exclusive breastfeeding and LAZ scores.

In this study, it was found that the socioeconomic level had an impact on LAZ scores, with an increase of 0.09 for each level of socioeconomic status which was measured by wealth quintiles. Many studies, such as Darteh et al.'s study in Ghana found that household wealth was related to stunting, where the lowest wealth quintiles had the lowest LAZ scores (41). However, this study was carried out on children under 5 years in Ghana. The underlying cause of low LAZ scores in Central Java among children under 2 years was the socioeconomic status and mother's education.

The implication of the results indicated that the nutritional status of women at child-bearing age has to be improved as studies had shown that macronutrient and food supplementation (42) as well as multiple micronutrient supplementation (43) had decreased low birth weight and increased birth outcomes. Education of the mothers on the feeding practice for their children under 2 years is also an important intervention. Therefore, intervention programs to prevent low birth weight and stunting should be done more effectively and efficiently.

The strength of this study was a relatively large sample size and the use of path analysis to measure the direct and indirect effect size and the total effect size of determinants to LAZ scores. The other benefits of using path analysis are the fact that the effect can be measured according to the conceptual framework. One of the findings in this study is that socioeconomic status was a relatively strong underlying determinant of LAZ scores. Thus, public health actions in the community that have low socioeconomic status to prevent stunting have to be performed, and one of them is through the family empowerment program. The limitation of the study was the shortness of the available variables in the 2021 ISSN survey. This cross-sectional design of this survey implied that the relations observed in path analysis cannot be taken as casual, and the direction could be reversed.

## 5. Conclusion

It is concluded that direct determinants of LAZ scores among children aged 6–23 months in Central Java, Indonesia, were birth length Z scores, the mother's age, exclusively breastfed history, and consumption of empty-calorie drinks. The underlying determinants were socioeconomic status and the mother's education. To prevent stunting among children aged 6–23 months in Central Java, Indonesia, intervention programs to increase the nutritional status of women at child-bearing age as well as education on child feeding practices should be conducted more efficiently and effectively.

## Data availability statement

The original contributions presented in the study are included in the article/supplementary files, further inquiries can be directed to the corresponding author/s.

## Ethics statement

The studies involving human participants were reviewed and approved by Health Research Ethics Committee of Public Health Faculty of Diponegoro University (134/EA/KEPK-FKM/2022). Written informed consent to participate in this study was provided by the participants' legal guardian/next of kin.

## Author contributions

MK, AS, SSuy, SSun, MI, NN, YN, JD, EW, MS, PH, and NF were responsible for the conception and design of the study. MK, AS, SSuy, and SSun were responsible for managing and retrieving the data. MK, AS, and SSun performed the statistical analysis and interpretation of data. MK wrote the manuscript. MK and AS did a critical review. All authors have read and approved the manuscript.
